# Imaging diagnostic standard in multiple endocrine neoplasia (MEN)

**DOI:** 10.1186/1470-7330-14-S1-P31

**Published:** 2014-10-09

**Authors:** J Tesdorff, T Wilhelm

**Affiliations:** 1 Department of Radiology, German Cancer Research Center, Heidelberg, Germany

## Background

Among all hereditary cancer syndromes MEN Type 1 and 2 are characterized by the concurrent but independent appearance of benign as well as malignant tumours. Neoplasias of parathyroid glands, pancreas, pituitary gland as well as neuroendocrine tumours of the stomach and intestinal wall are typical for MEN 1. Medullary thyroid carcinomas together with parathyroid adenomas and phaeochromocytomas are hallmarks of MEN 2. This presentation gives an overview about the multitude of imaging techniques that are inevitable for diagnostics and long-term follow up in MEN patients beyond molecular genetic and laboratory methods.

## Methods

Ultrasound (US) combined with nuclear medicine techniques are the leading methods to screen for pathologies of thyroid, parathyroid glands and cervical lymph nodes. For assessing metastases of a medullary thyroid carcinoma (MEN 2) DOTA-PET/CT is useful in addition to a CT/MR scan and neck and abdominal US. To detect phaeochromocytoma in MEN 2, CT and MRI are superior to US. MIBG scintigraphy can be performed in unclear cases. In primary hyperparathyroidism in MEN 1 cervical US is the leading method, supplemented by Tc-99m sestamibi scintigraphy or 11c-methionine PET/CT. DOTATOC PET/CT may supplement contrast-enhanced CT or MRI in detecting even small gastrinomas or other neuroendocrine tumours of the GI tract. In the examination of the pituitary gland, gadolinium enhanced dynamic MRI is standard.

## Conclusion

Given the complexity of multimodal imaging, a close collaboration of clinical radiology and nuclear medicine is essential to tailor the imaging protocol for MEN patients.

